# Regulatory elements of *Caenorhabditis elegans* ribosomal protein genes

**DOI:** 10.1186/1471-2164-13-433

**Published:** 2012-08-28

**Authors:** Monica C Sleumer, Guifeng Wei, Yunfei Wang, Hao Chang, Tao Xu, Runsheng Chen, Michael Q Zhang

**Affiliations:** 1 Bioinformatics Division, Center for Synthetic and Systems Biology, Tsinghua National Laboratory for Information Science and Technology, Tsinghua University, Beijing, China; 2 Laboratory of Noncoding RNA, Institute of Biophysics, Chinese Academy of Sciences, Beijing, China; 3 Department of Molecular and Cell Biology, Center for Systems Biology, University of Texas at Dallas, Richardson, TX, USA; 4 National Laboratory of Biomacromolecules, Institute of Biophysics, Chinese Academy of Sciences, Beijing, China

## Abstract

**Background:**

Ribosomal protein genes (RPGs) are essential, tightly regulated, and highly expressed during embryonic development and cell growth. Even though their protein sequences are strongly conserved, their mechanism of regulation is not conserved across yeast, *Drosophila*, and vertebrates. A recent investigation of genomic sequences conserved across both nematode species and associated with different gene groups indicated the existence of several elements in the upstream regions of *C. elegans* RPGs, providing a new insight regarding the regulation of these genes in *C. elegans*.

**Results:**

In this study, we performed an in-depth examination of *C. elegans* RPG regulation and found nine highly conserved motifs in the upstream regions of *C. elegans* RPGs using the motif discovery algorithm DME. Four motifs were partially similar to transcription factor binding sites from *C. elegans*, *Drosophila*, yeast, and human. One pair of these motifs was found to co-occur in the upstream regions of 250 transcripts including 22 RPGs. The distance between the two motifs displayed a complex frequency pattern that was related to their relative orientation.

We tested the impact of three of these motifs on the expression of *rpl-2* using a series of reporter gene constructs and showed that all three motifs are necessary to maintain the high natural expression level of this gene. One of the motifs was similar to the binding site of an orthologue of POP-1, and we showed that RNAi knockdown of *pop-1* impacts the expression of *rpl-2*. We further determined the transcription start site of *rpl-2* by 5’ RACE and found that the motifs lie 40–90 bases upstream of the start site. We also found evidence that a noncoding RNA, contained within the outron of *rpl-2*, is co-transcribed with *rpl-2* and cleaved during trans-splicing.

**Conclusions:**

Our results indicate that *C. elegans* RPGs are regulated by a complex novel series of regulatory elements that is evolutionarily distinct from those of all other species examined up until now.

## Background

Ribosomes are essential components of all cells, prokaryotic and eukaryotic, and the sequences of ribosomal protein genes (RPGs) are conserved across all eukaryotes. However, the regulation of expression of RPGs has seldom been studied; in fact, they are often excluded from gene regulation experiments because they do not normally display tissue-specific differential expression. Regulation of RPGs is important because their expression is regulated very precisely: each ribosome contains exactly one each of up to 84 different proteins, and errors in the expression levels of these genes will result in malformed ribosomes [1]. Because ribosomes are necessary for the expression of all protein-coding genes, they are highly expressed in replicating cells. RPG expression levels are rate-limiting on cell growth [2], and their overexpression is required for the proliferation of cancer cells [3].

RPG regulation has been studied in several species including yeast, *Drosophila*, and mammals. In yeast, RPGs are generally regulated by a combination of transcription factors (TFs) Rap1p, Fhl1p, Ifh1p, and sometimes Abf1p, Cbf1p, Hmo1p, Sfp1p, Crf1p, or Tbf1p, but the exact combination varies widely from species to species [4-7]. In *Saccharomyces cerevisiae*, most RPGs are regulated via the Target of Rapamycin pathway, in which Rap1p and Fhl1p bind to sites in RPG promoters. Coactivator Ifh1p binds to Fhl1p to upregulate expression during periods of rapid cell growth, while phosphorylated Crf1p, a corepressor, binds to Fhl1p to downregulate expression during conditions that are unfavourable for growth [7,8]. Additionally, the functionality of the archaic Homol-D element, whose binding protein is currently unknown, remains essential for the regulation of RPG expression in eight yeast species, but has been displaced entirely by Rap1p in another six yeast species [5]. In a recent review, Weirauch and Hughes assessed evidence showing that the TFs (and TF binding sites) responsible for regulation of RPGs were very different between *Saccharomyces cerevisiae* and *Candida albicans*, and that the distribution of the binding sites with respect to the genes’ transcription start sites (TSSs) was both dependent on which TFs were involved and highly similar across the upstream regions of RPGs within each species [9]. Furthermore, numerous yeast RPGs exist in two copies, and Zeevi et al. recently showed that the promoters of single copy RPGs have a higher expression level than those of dual-copy RPGs to preserve the correct stoichiometry [10].

In *Drosophila*, RPG promoters contain a poly-pyrimidine sequence just upstream of the translation start site (ATG), binding sites for the DNA replication-related element factor (Dref) and Nf1, as well as two motifs of unknown functional mechanism, one of which is similar to the Homol-D element in the upstream region of some yeast RPGs [11]. The Dref binding site was found to occur within 600 base pairs (bps) of the TSS in the majority of cases [11]. These characteristics of RPG promoters were found to be common to all species of *Drosophila* studied. However, the specific sequences and motif locations varied widely from species to species, suggesting a high rate of binding site turnover under the condition of module-wise stabilizing selection [11]. Similar to *Drosophila*, the promoters of human and other mammalian RPGs also contained polypyrimidine tracts at the TSS and binding sites for ZBED1, the human homologue of Dref (Note that the ZBED1 binding site is referred to by Perry as “Box A” and by Yamashita et al. as “hDRE”) [12,13]. The ZBED1 binding sites displayed an even stronger location bias in human RPG promoters than they did in *Drosophila*, with 20/22 predicted ZBED1 binding sequences in the range 11 to 73 bp upstream of the the putative TSS [13]. Mammalian RPG promoters also contained TATA boxes and binding sites for GABP, SP1, and YY1, which were not found in *Drosophila* RPG promoters, but were evolutionarily conserved in the RPG promoters of other vertebrates such as amphibians and fish [12].

Taken together, these studies show that while the precise stoichiometric expression of RPGs is conserved across all species, the specific mechanism by which this regulation is achieved is often not conserved (even among closely related species), implying that it evolves much more quickly than the genes themselves [11]. The overlap between regulatory elements of RPGs among all species studied thus far is very weak, suggesting that nematodes may possess yet another mechanism of RPG regulation. Additionally, most protein-coding transcripts in *C. elegans* are trans-spliced, a process during which the original 5’ UTR (the “outron”) is replaced by a standardized 22 bp sequence just upstream of the ATG, providing a mechanism for gene regulation not found in most other eukaryotes. An investigation into how *C. elegans* RPGs are regulated could lead to further insights applicable to both systems and evolutionary biology.

Given its extensive history as a model organism in the field of genetics, surprisingly little is known about gene regulation in *C. elegans*. The regulation of most genes remains poorly understood, and although 934 TFs have been identified in the *C. elegans* genome [14], the binding specificities and *in vivo* binding sites of all but a few of these TFs remains undescribed. Attempts to find novel TF binding sites purely by comparative genomic analysis were stymied by the remarkable similarity of the intergenic regions of different *Caenorhabditis* species in spite of their long evolutionary distance [15].

In a recent investigation, we searched for elements that were conserved across the promoters of not only orthologous genes in several nematode genomes, but also functionally related genes in *C. elegans*[16]. Although that study did not focus on any particular set of genes *ab initio*, the primary result was the discovery of a set of eight novel elements that were associated with *C. elegans* RPG promoters. Together, the eight motifs appeared in the upstream regions of 63 annotated RPGs in the *C. elegans* genome. Three of the eight motifs were similar to previously characterized TF binding sites in other species, but the other five were not similar to any known genomic elements. Six of the motifs showed a location bias in the region 200–400 bp upstream of the RPGs, and preliminary findings also suggested that the motifs had a specific co-distribution with respect to the distance between the different motifs [16].

These findings, while preliminary, implied that *C. elegans* RPGs possess a unique system of regulation, and that their genomic environment contains numerous specific elements. We expect further investigation of RPG-associated genomic elements to lead to a deeper understanding of gene regulation in general and regulation of RPG expression in particular, specifically the significance of the spatial distribution of genomic elements with respect to the TSS, the trans-splice acceptor site, and the ATG. Here, we endeavoured to discover more about the regulation of *C. elegans* RPGs by performing a comprehensive investigation of conserved motifs in RPG promoters. Specifically, we wanted to determine what motifs were over-represented in *C. elegans* RPG upstream regions compared to the upstream regions of other protein-coding genes, and then determine the functions of the motifs, especially with respect to their impact on RPG expression regulation. We hypothesized that an RPG-focused motif analysis would rediscover at least some of the motifs described in Sleumer et al. [16]. We further proposed that many of the motifs would function as TF binding sites, but some may be transcribed and function at the RNA level, while others may have a structural function in the DNA double helix. We expected that if the TF-binding motifs were removed or mutated, the regulation of the genes would be impacted, and (based on preliminary experiments described in Sleumer et al. [16]) the gene expression level would decrease.

Previous results indicated that most *C. elegans* RPG-associated motifs were found approximately 300 bp upstream of the ATG [16], therefore we extracted the upstream region of each transcript up to the end of the next protein-coding gene or a maximum of 700 bp. We used DME to find conserved motifs in the upstream regions of 84 identified cytoplasmic *C. elegans* RPGs [17,18]. DME is a program that finds over-represented short sequences and sequence variations in a sequence set with respect to a background sequence set. We then analyzed the motifs with respect to their similarity to known TF binding sites, distribution with respect to the ATG, distribution across the upstream regions of all protein-coding genes, and mutual co-occurrence.

We used 5’ RACE experiments and Green Fluorescent Protein (GFP) expression constructs to determine the TSS of *rpl-2* and test the impact on gene expression of three motifs in its upstream region. The motif with the strongest impact on *rpl-2* expression was similar to the binding site of an orthologue of POP-1, so we knocked down *pop-1* with RNAi and showed that the expression of *rpl-2* was negatively affected.

In total we discovered nine RPG-associated motifs, of which four were similar to known TF binding sites, two were novel, two were related to AA/TT dinucleotide hyperperiodicity, and one overlapped trans-splice acceptor sites. We determined that one pair of motifs co-occurred in a noteworthy co-distribution pattern. We found that the TSS was a short distance downstream of the three motifs, discovered evidence that *rpl-2* may be co-transcribed with a ncRNA in its upstream region, and showed that all three motifs were necessary for the effective expression of *rpl-2*.

## Results

### Motif discovery

**Figure 1:**
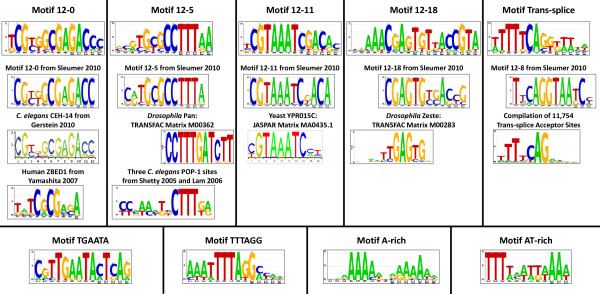
**Motif logos.** Logos of the nine motifs found in the current work, aligned with comparable logos from the literature where applicable.

**We detected nine motifs in the immediate upstream regions of *C. elegans *cytoplasmic RPGs.** We identified 84 *C. elegans* cytoplasmic ribosomal protein transcripts from the Ribosomal Protein Gene Database [18]. We extracted the upstream regions of the RPGs and used the motif discovery algorithm DME to find motifs using the set of all upstream regions as a background [17]. We identified nine significant motifs that each appeared upstream of between 16 and 81 of the RPGs (Table 1; Figure 1). Five of the motifs (12–0, 12–5, 12–11, 12–18, and Trans-splice) were clearly similar to motifs we observed in a previous analysis using entirely different input data (Figure 1) [16]. Four of these motifs (all but Trans-splice) displayed a location bias, consistently occurring in a single instance between 200 and 500 bp upstream of the ATG.

**Table 1:** List of nine motifs discovered in the upstream regions of RPGs

**Name**	**Num DME results**	**Num RPG instances**	**Num RPG seq**	**Upstream distribution range**	**Num total instances**	**Num total sequences**	**Fold enrichment**	**Notes**
12-0	3	36	36	238-456	903	752	10.64	Co-occurrence with 12–5; Similar to CEH-14 binding site
12-5	3	27	27	253-503	884	775	8.15	Co-occurrence with 12–0; Similar to Pan binding site
12-11	3	18	18	227-464	493	383	9.75	Similar to YPR015C site
12-18	3	26	24	154-665	426	416	16.30	Similar to Zeste binding site
TGAATA	2	17	16	14-609	86	85	52.78	Novel
TTTAGG	2	39	34	71-586	1383	1283	7.53	Novel
A-rich	4	475	81	52-650	76,172	19,169	1.66	WWN_6_WW
AT-rich	2	96	45	30-628	13,096	7223	1.96	WWN_6_WW
Trans-splice	3	64	52	0-579	3860	3351	4.43	Half of instances overlap trans-splice acceptor sites

Column “Name” shows the motif name; “Num DME results” shows the number of DME results that were merged to form the final motif; “Num RPG instances” shows the number of sites among RPG upstream regions; “Num RPG seq” shows the number of RPG upstream regions that contain the motif; “Upstream distribution range” shows the 95% distribution range (in bp) of the motif with respect to the ATG; “Num total instances” indicates the total number of sites of the motif found in all 22,428 upstream regions by ModuleMaster [32]; “Num total sequences” indicates the number of upstream regions that contain at least one instance of each motif according to ModuleMaster; “Fold enrichment” indicates the fold enrichment of the motif in RPG upstream regions compared to all upstream regions; “Notes” indicates any other pertinent information.

Two motifs (A-rich and AT-rich) were characterized by one or two highly conserved AT base pairs followed by a poorly conserved portion of six base pairs and then another one or two highly conserved AT base pairs. In spite of the high frequency of this pattern in the AT-rich *C. elegans* genome, both of these motifs were significantly over-represented in the set of ribosomal upstreams and were found to be uniformly distributed. Two other motifs (TGAATA and TTTAGG) were novel and displayed a tendency to have only one instance per sequence, although they did not have a location bias.

About half of the instances of motif Trans-splice overlapped trans-splice acceptor sites. Wormbase *C. elegans* genome version WS220 contained 12,890 unique trans-splice acceptor sites, of which 125 (0.97%) occurred in RPG upstream regions; given that there were only 84 RPG upstream regions in our total set of 22,428 (0.37%), this a 2.6-fold enrichment over the background level. The over-representation of trans-splice acceptor sites among RPG upstream regions may explain why a trans-splice acceptor site-like motif appeared in our motif discovery results.

### Motif co-occurrence among RPGs

**Figure 2:**
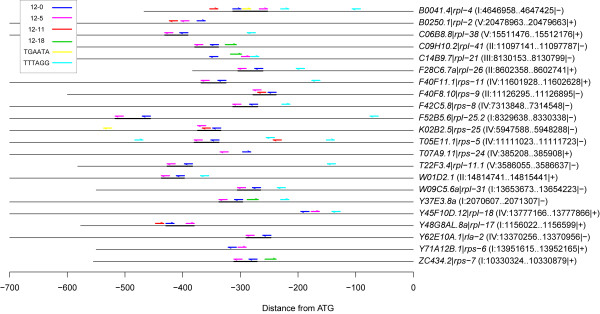
**Motif distribution in the 22 RPG upstream regions containing both 12–0 and 12–5.** Each upstream region is represented by a horizontal line, with the ATGs of the transcripts aligned at the right edge of the figure. Locations of motifs 12–0, 12–5, 12–11, 12–18, TGAATA, and TTTAGG are shown in dark blue, magenta, red, green, yellow, and cyan respectively. Arrows indicate motif strand. The 17 motif pairs in the 12–0 ⇛ 12–5 orientation are indicated by black bars.

**Motifs 12–0 and 12–5 displayed a significant and interesting co-occurrence pattern.** For each pair of motifs, we determined the significance of the number of RPG upstream regions containing both motifs compared to the number of upstream regions containing only one of the two motifs by the Fisher Exact test. One pair of motifs displayed significant co-occurrence: 22 RPGs contained both 12–0 and 12–5 in their upstream regions, even though the expected number of co-occurrences for these two motifs was 12 (based on their individual frequencies; Bonferroni-corrected p-value: 1.07E-04). The two motifs occurred between four and 42 bp apart on all 22 of these genes, and appeared in the same relative order and orientation, with motif 12–0 located 5’ to motif 12–5 (henceforth referred to as “12-0 ⇛ 12-5”), in 17 of these co-occurrences (Figure 2). The one-tailed p-value for this fraction by the binomial test is 4.00E-7 based on the assumption that only 1/4 of motif pairs would be in this orientation if they were randomly distributed. The other five motif pairs were in an alternate orientation with motif 12–5 on the opposite strand and 5’ to motif 12–0 (henceforth referred to as “R12-5 ⇛ 12-0”).

**Figure 3:**
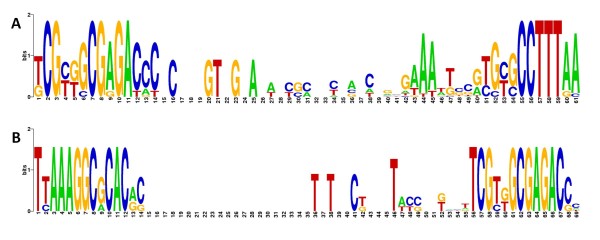
**Logos of 12–0 ⇛ 12–5 and R12-5 ⇛ 12–0 motif pairs in RPG upstream regions. A:** Logo of 17 aligned motif pairs upstream of RPGs in the 12–0 ⇛ 12–5 relative orientation. **B:** Logo of five aligned motif pairs upstream of RPGs in the R12-5 ⇛ 12–0 orientation.

To determine whether the intervening sequence contained any other conserved bases, we extracted and aligned the entire sequence encompassing both motifs from the 12–0 ⇛ 12–5 motif pairs (Figure 3A). We observed several conserved bases in the region between the two motifs, but no intervening motif. Similarly, we constructed a combined logo of the five R12-5 ⇛ 12–0 motif pairs and observed no intervening motif (Figure 3B).

Hajarnavis and Durbin made an interesting observation regarding the 3’ UTRs of *C. elegans* RPGs: 30 of the 84 ribosomal protein 3’ UTRs contained two instances of a UUGUU sequence on either side of the polyadenylation signal at the very end of the transcript [19].We evaluated the rate of co-occurrence between the motifs and the 30 RPGs with 3’ UTR elements, however we observed no relationship between their distributions.

### Comparison of motifs to known TF binding sites

**One motif was strongly similar to the *C. elegans *CEH-14 ChIP-Seq motif and weakly similar to the ZBED1 site; three other motifs were similar to TF binding sites from *Drosophila *and yeast.** Motif 12–0 was nearly identical to the CEH-14 motif, which was compiled from a set of 1170 ChIP-Seq peaks as part of the modENCODE project (Figure 1) [20,21]. The CEH-14 peaks, like the 12–0 sites, were strongly associated with RPG upstream regions: of the 84 RPG upstream regions, 57 overlapped with a CEH-14 ChIP-Seq peak (p < 2.2E-16), and of these, 23 overlapped with a 12–0 site.

We also observed a weak similarity between motif 12–0 and the 20 RPG-related binding sites of ZBED1 described by Yamashita et al. [13]: Both sites contained the core sequence GCGAGA, however, the ZBED1 binding sequence is palindromic while the sequence of 12–0 is not. The *C. elegans* genome contains a possible orthologue of ZBED1, *bed-3*, which is involved in regulation of lineage-specific cell division during vulval development [22]. We observed no other similarities between the motifs and the regulatory elements of RPGs in yeast, *Drosophila*, or vertebrates. Given the prominent GAGA sequence within motif 12–0, we compared it to known GAGA-factor binding sites. GAGA-factor binding sites are composed of either two or more GAG trinucleotides, or else a GA repeat of five or more bases, neither of which are present in the 12–0 sequence, therefore we concluded that 12–0 is not likely to be a GAGA-factor binding site [23].

All nine motifs were compared to known sites from a wide variety of TF binding sequence databases using three comparison methods: STAMP [24], Matcompare [25], and TESS [26]. Motif 12–5 was found by Matcompare to be similar to TRANSFAC TCF1-like matrix M00362, a binding site of the *Drosophila* HMG box-containing factor Pan [27,28]. *C. elegans* has one TCF-family TF called POP-1. Motif 12–11 was found by STAMP to be similar to the binding site of YPR015C (JASPAR accession number MA0435.1), a yeast C2H2 zinc finger TF of unknown function whose overexpression causes cell cycle delay or arrest [29,30]. A BLASTP of the protein sequence of this gene against the *C. elegans* genome yielded many similarly-scored matches against the numerous C2H2 zinc finger domains in the *C. elegans* proteome, therefore it is unknown whether *C. elegans* has a true orthologue of this gene. Motif 12–18 was found by TESS to be similar to the binding site of *Drosophila* Zeste (TRANSFAC Matrix M00283). This finding was consistent with the finding by Sleumer et al. [16] that motif 12–18 from that publication, which is very similar to motif 12–18 of the current work, is also similar to the binding site of *Drosophila* Zeste. *C. elegans* has one orthologue of Zeste, MES-2, whose binding specificity is currently unknown.

### TATA box scan

**RPGs are no more likely than other genes to contain a TATA box-like sequence in their upstream region.** Due to the lack of a TATA box-like motif among the motif discovery results, we investigated the genomic distribution of TATA-boxes in *C. elegans*, which are poorly characterized and obscured by trans-splicing. Berendzen et al. [31] showed that only one main TATA box-related hexamer, TATAAA, was overrepresented in *C. elegans* core promoters, and that the distribution of this hexamer displayed a peak between 30 and 80 bp upstream of the ATG on the same strand. Therefore, we scanned the upstream regions of all protein-coding transcripts for instances of the sequence TATAAA. We found that even though the sequence occurred slightly more frequently in the core promoter than elsewhere in the genome, in general this TATA box sequence was quite rare; only 1951 of 22,428 *C. elegans* protein-coding transcripts (8.7%) contained a TATAAA sequence in the specified region. In comparison, four of the 84 RPGs (4.8%) contained a TATAAA sequence in the same region. The Fisher Exact p-value for this distribution is 0.25, indicating that although very few *C. elegans* RPG upstream regions had TATA-boxes, they did not have TATA-boxes at a significantly lower rate than genes in general.

### Motif distribution across all upstream regions

ModuleMaster is a program that can scan sequences for individual or combinatorial matches to position weight matrices using the MATCH matrix scan algorithm [32,33]. Here, we used ModuleMaster to scan the upstream regions of all protein-coding transcripts for instances of the nine discovered motifs, with the goal of determining the distribution of the motifs in the upstream regions of transcripts other than RPGs. The number of upstream regions that contained each motif varied widely from 85 to over 19,000 (Table 1; Additional file 1).

We wondered whether motifs A-rich and AT-rich might be related to nucleosome coverage. To investigate this question, we compared the distributions of these motifs with genome-wide nucleosome coverage scores [34]. We found a weak negative correlation between the number of A-rich or AT-rich motifs per base of upstream region and the average nucleosome coverage (r = −0.245 and r = −0.154 respectively).

**Motif 12–0 was strongly associated with CEH-14 ChIP-Seq peaks.** Motif 12–0 occurred upstream of 752 transcripts, of which 141 also overlapped a CEH-14 peak. Given the individual occurrences of motif 12–0 and CEH-14 peaks, the expected number of overlaps was only 38; the p-value for this high number of overlaps was less than 2.2E-16 by the Fisher Exact test.

### Motif conservation in other nematode genomes

**Figure 4:**
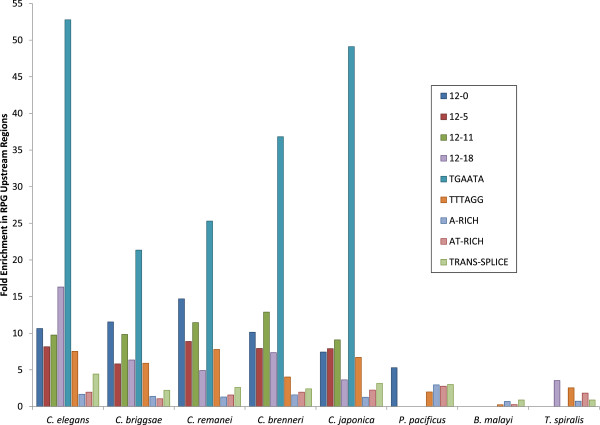
**Fold enrichment of all nine motifs in eight nematode genomes.** Graph showing the fold enrichment of each of the nine motifs in the upstream regions of RPGs compared to those of all protein-coding genes for eight species of nematodes. The values for *C. elegans* are the same as those shown in Table 1.

**The motifs were conserved among species in genus *Caenorhabditis***, **but not in other species of nematodes.** We obtained orthologues of *C. elegans* protein-coding genes from seven other assembled nematode genomes as previously described and used ModuleMaster to scan the upstream regions of all orthologues for instances of each of the nine motifs [15,32]. We then calculated the fold enrichment of instances of each motif among RPG upstream regions compared to all upstream regions (Figure 4). We found that the other four species in genus *Caenorhabditis* had similar enrichment values as *C. elegans*, indicating both that they possess numerous instances of each motif and that the motifs are specifically enriched in RPG upstream regions. However the other three species examined (*Pristionchus pacificus*, *Brugia malayi*, and *Trichinella spiralis*) showed no significant enrichment for any of the motifs. In fact, with the exception of two instances of 12–0 in *P. pacificus* and two instances of 12–18 in *T. spiralis*, none of these species had any instances of the first five motifs in their RPG upstream regions at all. For the four remaining motifs, *P. pacificus* displayed slight enrichment, while *T. spiralis* and *B. malayi* displayed either no significant enrichment or else net depletion.

### Motif co-occurrence among all upstream regions

**Fourteen pairs of motifs co-occurred across all upstream regions.** All pairs of motifs (including same-strand occurrences of TATAAA as a tenth motif), were analyzed with respect to the number of upstream regions in which they co-occurred compared to the number of upstream regions containing only one motif or neither motif. We used the Fisher Exact Test to determine the significance of the values, by way of a Bonferroni-adjusted p-value threshold of 0.0002 (0.01/45 comparisons) (Table 2). Fourteen pairs of motifs co-occurred in a significantly higher than expected number of upstream regions based on the individual occurrences of the motifs, and two pairs had a lower-than-expected co-occurrence.

**Table 2:** Motif co-occurrence

**Comparison**	**Upstreams containing first motif only**	**Upstreams containing second motif only**	**Upstreams containing neither motif**	**Intersection (upstreams containing both motifs)**	**P-value**	**Expected intersection**	**Intersection/ Expected intersection**	**Notes**
12-0 and 12-5	502	525	19794	250	8.2E-178	28	9.04	Includes 12-0
A-rich and TATAAA	11261	1251	651	7908	1.4E-93	8332	0.95	Anti-occurrence
12-0 and 12-11	684	315	20004	68	1.9E-28	14	4.97	Includes 12-0
12-11 and 12-5	328	720	19968	55	3.9E-18	14	3.90	
12-5 and AT-rich	411	6859	13437	364	1.3E-13	266	1.37	
12-0 and TTTAGG	664	1195	19124	88	3.4E-09	46	1.92	Includes 12-0
12-0 and Trans-splice	575	3174	17145	177	3.5E-08	120	1.48	Includes 12-0
12-0 and AT-rich	423	6894	13425	329	4.9E-08	258	1.28	Includes 12-0
12-5 and Trans-splice	596	3172	17124	179	1.0E-07	123	1.45	
AT-rich and Trans-splice	5951	2079	11769	1272	1.27E-06	1149	1.11	
12-0 and 12-18	716	380	19939	36	1.2E-06	15	2.42	Includes 12-0
TTTAGG and Trans-splice	1017	3085	16703	266	2.2E-06	204	1.30	
AT-rich and TTTAGG	6707	767	13081	516	5.2E-06	440	1.17	
A-rich and AT-rich	12678	732	1170	6491	6.2E-05	6571	0.99	Anti-occurrence
12-5 and TTTAGG	701	1209	19087	74	1.2E-04	47	1.57	
12-18 and 12-5	385	744	19911	31	1.9E-04	15	2.03	

Out of all 22,428 upstream regions, 21,071 contained at least one instance of one motif or the same-strand TATAAA sequence. For each pair of motifs (including TATAAA), the upstream regions were divided into four categories: Upstream regions that contained the first motif but not the second, upstream regions that contained the second motif but not the first, upstream regions that contained neither of the two motifs, and upstream regions that contained both motifs (the intersection). We calculated the probability of this distribution by the Fisher Exact Test. We also calculated the expected intersection, based on the individual frequency of each motif, and then calculated the ratio of the actual intersection to the expected intersection to show whether the motif pairs co-occurred more or less frequently than expected. Shown are those motif pairs whose p-values of co-occurrence were more significant than a Bonferroni-adjusted threshold of 0.0002 (0.01/45 comparisons). Motif pairs that include motif 12–0 and motif pairs that co-occurred in fewer upstream regions than expected are indicated in column “Notes”.

Given that only one pair of motifs displayed significant co-occurrence among the RPG upstream regions, the number of co-occurring pairs of motifs across all upstream regions was surprisingly high. Motif 12–0 significantly co-occurred with six of the nine other motifs. Just as for the RPG upstream regions, motifs 12–0 and 12–5 displayed the strongest co-occurrence tendency; the two motifs had similar individual frequency counts and co-occurred for about one-third of their instances. Many of the co-occurrence values were only marginally different from the expected value, but because the numbers were so large, the probability of seeing such a deviation by chance was low.

Two comparisons showed a co-occurrence pattern that was significantly lower than expected: A-rich with TATAAA, and A-rich with AT-rich. This was surprising and unexpected; because A-rich, AT-rich, and TATAAA sequences can all overlap, we would have anticipated them to have a higher-than-expected co-occurrence value.

### Co-distribution of motifs 120 and 125

**Figure 5:**
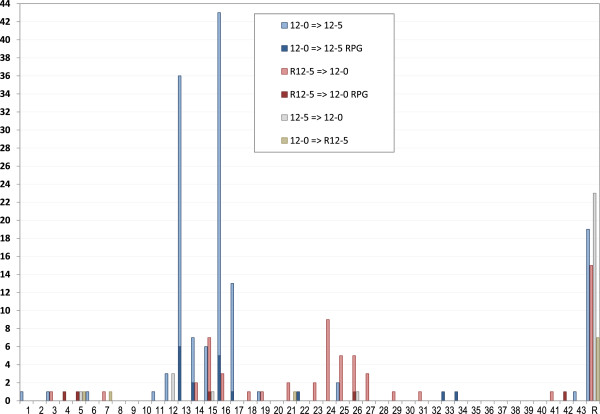
**Distance between motifs 12–0 and 12–5 in all four orientations.** Histogram of the distance between motifs 12–0 and 12–5 for all upstream regions that contained both motifs. Blue bars: Distribution of the inter-motif distance for motif pairs in the 12–0 ⇛ 12–5 orientation. The 17 pairs in this category among RPG upstream regions are indicated by the dark blue portion of each bar. Red bars: Distribution of the inter-motif distance for motif pairs in the R12-5 ⇛ 12–0 orientation. The five pairs in this category among RPG upstream regions are indicated in dark red. Grey bars: Distribution of the inter-motif distance for motif pairs in the 12–5 ⇛ 12–0 orientation. Brown bars: Distribution of the inter-motif distance for motif pairs in the 12–0 ⇛ R12-5 orientation. “R” indicates the remaining motif pairs in each category with distances greater than 43 bp.

**Motifs 12–0 and 12–5 displayed a complex co-distribution pattern consisting of two preferred relative orientations with different inter-motif spacing.** Following our finding that motifs 12–0 and 12–5 were between four and 42 bp apart on all 22 RPG upstream regions that contained both motifs, we generated a histogram of the distances between the two motifs for all upstream regions that contained both motifs (Figure 5). Of the 250 upstream regions that contained both motifs (Table 2), 60 were on bidirectional promoters and 26 contained more than one instance of either 12–0 or 12–5 (or both), resulting in a total of 240 inter-motif distances. One hundred thirty-eight (58%) motif pairs occurred in the 12–0 ⇛ 12–5 orientation, of which 119 (86%) had a distance of less than 44 bp. Another 63 (26%) motif pairs were in the R12-5 ⇛ 12–0 orientation, of which 48 (76%) were less than 44 bp apart. Only 39 (16%) motif pairs appeared in one of the remaining two orientations.

The distance frequency distributions of the 12–0 ⇛ 12–5 and R12-5 ⇛ 12–0 motif pairs were markedly different. Almost all of the 12–0 ⇛ 12–5 motif pairs were between 12 and 17 bp apart, with sharp peaks at 13 and 16 bp apart. Conversely, the R12-5 ⇛ 12–0 motif pairs displayed a bimodal distribution with peaks at 15 and 24 bp apart. The distances between motif pairs that were more than 43 bp apart displayed a long flat distribution.

**Figure 6:**
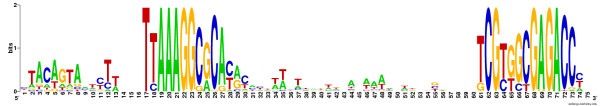
**Logo of R12-5 ⇛ 12–0 with flanking motif.** Logo of the 42 aligned motif pairs in the R12-5 ⇛ 12–0 orientation that were separated by 14 to 31 bp, including the 5’ flanking region. Twenty of these pairs contained the sequence TACWGTA within nine bp of the beginning of motif 12–5.

**Motif pair R12-5 ⇛ 12–0 possessed an additional conserved TACWGTA sequence in the flanking region.** We examined the intermotif and flanking sequences of all 12–0 ⇛ 12–5 and R12-5 ⇛ 12–0 motif pairs. We observed that of the 42 R12-5 ⇛ 12–0 motif pairs separated by 14 to 31 bp, 20 contained the palindromic sequence TACWGTA within nine bp of the beginning of R12-5 (Figure 6).

### DAVID analysis

**Motifs 12–0 and 12–5 were associated with genes involved in cell-cycle processes and reproductive development.** We used DAVID to determine whether genes whose upstream regions contained any given motif were also significantly associated with specific GO terms and other functional annotations (Additional file 2) [35,36]. DAVID is a web-based program that can examine a list of genes for enrichment of GO terms and metabolic pathway membership. When all genes associated with a motif were included in the DAVID analysis, we observed that the motifs, with the exceptions of motifs A-rich and AT-rich, were strongly associated with ribosomal proteins and related categories. Of these, all but 12–18 and TGAATA were associated with other RPGs in addition to the minimal set of 84 that was used for motif discovery. The most significant association was between motif 12–0 and the GO Cellular Component term “Ribonucleoprotein Complex”, which had a Benjamini-corrected p-value of 2.5E-25. Motifs 12–0 and 12–5 were also associated with the GO Biological Process category “Embryonic Development Ending in Birth or Egg Hatching”, which was a superset of the ribosomal, cell cycle, sex differentiation, etc. categories and contained 3322 *C. elegans* gene products. Motifs A-rich and AT-rich were significantly associated with vague categories such as “Alternative splicing” and “Transmembrane” from the Protein Information Resource (PIR) [37].

In order to determine which gene categories other than RPGs were associated with the motifs, we excluded the 84 cytoplasmic RPGs from the gene lists and repeated the DAVID analysis. Motifs 12–0, 12–5, and 12–11 had significant associations with GO Biological Process terms such as “Embryonic Development Ending in Birth or Egg Hatching”, “Reproductive Developmental Process”, and “Cell Cycle Process”, indicating that these three motifs were associated with other important genes in addition to RPGs. The remaining motifs were not associated with interesting gene categories after RPGs were removed from the input set.

The set of 228 genes with upstream regions containing both 12–0 and 12–5 (excluding RPGs; 209 genes mapped by DAVID) was significantly associated with the following GO terms: “Reproductive Developmental Process” (Benjamini-corrected p-value 1.1E-3), “Hermaphrodite Genitalia Development” (p-value 8.7E-3), “Embryonic Development Ending in Birth or Egg Hatching” (p-value 7.3E-3), “Sex Differentiation” (p-value 1E-2), and “Germline Cell Cycle Switching, Mitotic to Meiotic Cell Cycle” (p-value 4.0E-2). When the list was reduced to those upstream regions in which the two motifs were within 44 bp of each other, or those in which the two motifs were within 44 bp and in the 12–0 ⇛ 12–5 orientation, the same categories were seen, and the significance increased slightly.

### Impact of motifs on gene expression

We generated a series of GFP expression constructs to test the impact of the motifs on gene expression of RPGs. We chose gene *B0250.1* (*rpl-2*) as a testing candidate because its upstream region contained only three motifs, 12–0, 12–5, and 12–11, other than instances of A-rich, AT-rich, and Trans-splice. The three motifs occurred close together, in the respective locations 362, 390, and 410 bp upstream of the ATG, and were therefore testable as a group (Additional file 3). The first objective of the gene expression experiments was to determine the location of the TSS for this gene and thereby the distance between the TSS and the motifs. This was necessary in order to establish whether the motifs were downstream of the TSS and may function at the RNA level, or whether the motifs were not transcribed and thus may function at the DNA level. The second objective was to determine whether the motifs were necessary for gene expression.

**The TSS of *rpl-2 *was 322 bp upstream of the ATG.** We used 5’ RACE to determine the TSS of *rpl-2*. This experiment was confounded by two factors: the presence of ncRNA *B0250.15* in the immediate upstream region of *rpl-2* between the motifs and the ATG [20], and the trans-splicing of *rpl-2*, which meant that the original 5’ UTR was not reliably detectable by ordinary 5’ RACE (Additional file 3).

To overcome these issues, we first generated a GFP expression construct in which the region from 94 to 671 bp upstream of *rpl-2*, including the motifs but excluding the trans-splice acceptor site, was inserted into a GFP expression vector. We injected the plasmid into the gonad of young adult worms and established stable transgenic lines from GFP-expressing members of the F1 generation. We isolated total RNA from these lines and performed a 5’ RACE experiment on the transcript from the plasmid. We determined that the TSS of the non-trans spliced expression construct corresponded to the position 322 bp upstream of the ATG of *rpl-2* in the *C. elegans* genome. This location also corresponds to the position 10 bp downstream of the predicted start site of ncRNA *B0250.15* (Additional file 3). The three motifs 12–0, 12–5, and 12–11 were thereby found to occur 40, 68, and 88 bp upstream of the TSS respectively.

We further performed RT-PCR and were able to amplify the upstream region of *rpl-2* between the 5’ RACE adapter and the vector sequence, which included the entire length of ncRNA *B0250.15* other than the first 10 bp (Additional file 4). These results suggested that ncRNA *B0250.15* was co-transcribed with *rpl-2* and that the mature ncRNA was processed from the outron after trans-splicing had taken place.

**Figure 7:**
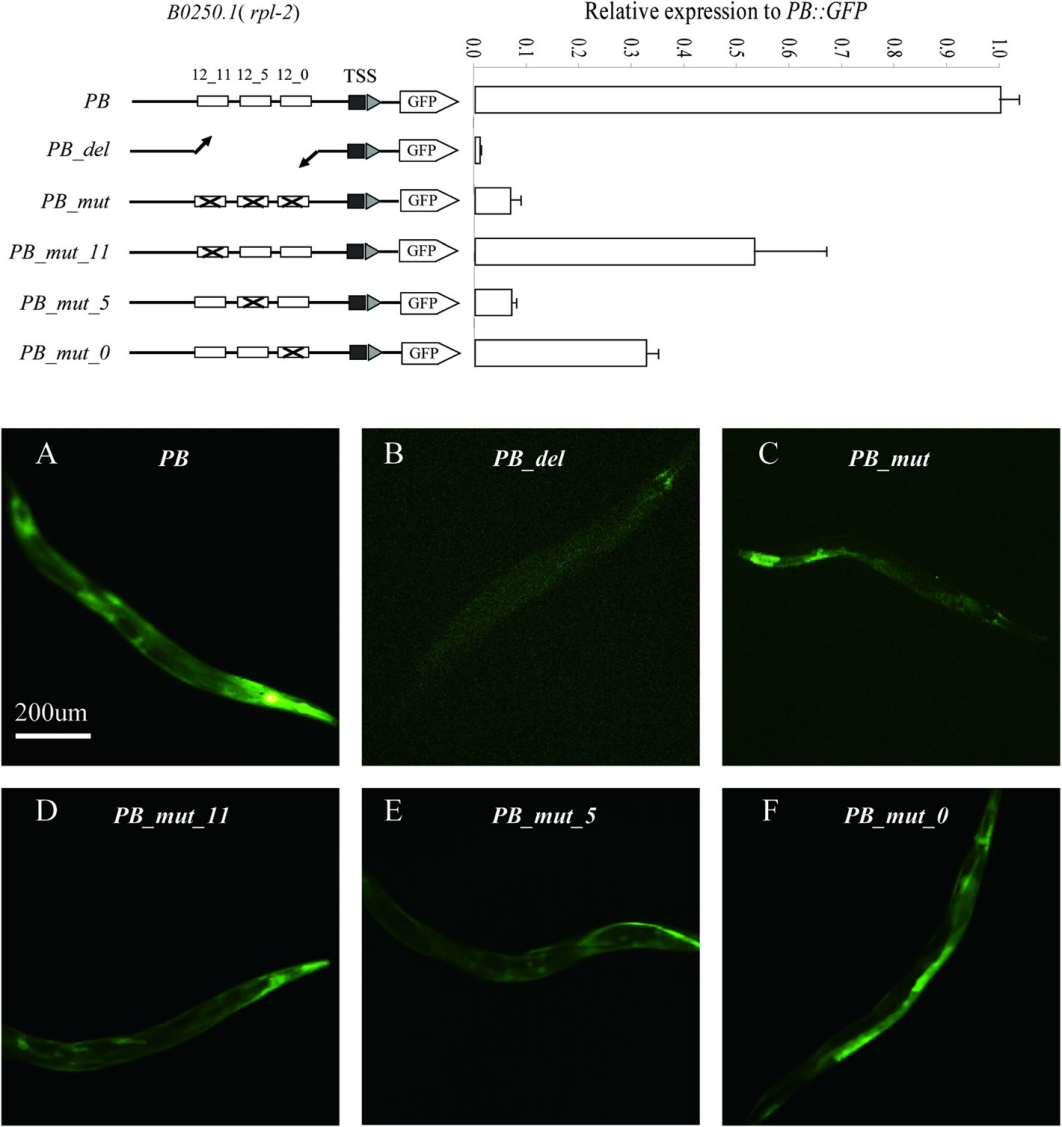
**GFP expression experiments to test motif function.** Top left: Schematic of the GFP expression constructs generated in this experiment (not to scale; for exact locations of all elements, see Additional file 3). The upstream region of gene *rpl-2* (*B0250.1*) contained instances of motifs 12–11, 12–5, and 12–0 close together, about 380 bp upstream of the ATG. We generated six expression constructs to test the impact of the motifs on the expression of *rpl-2* as follows: intact upstream region (PB); all three motifs excised (PB_del); all three motifs mutated (PB_mut); Motif 12–11 mutated (PB_mut_11); Motif 12–5 mutated (PB_mut_5); Motif 12–0 mutated (PB_mut_0). See Additional file 6 for all primers used to generate the constructs. Top right: Quantitative GFP expression signals were measured by fluorescent quantitative microscopy and scaled relative to that from worms carrying plasmid PB. Error bars indicate the standard error after four to seven measurements. Bottom: Photographs of *in vivo* expression of each construct.

**Motifs 12–0, 12–5, and 12–11 were all essential for native expression of *rpl-2***; **the absence of motif 12–5 had the greatest impact.** We generated six expression constructs for this gene: one which was the same as that described above, containing the intact upstream region from 94 to 671 bp upstream of the ATG attached to the GFP reporter gene, one in which the portion of the upstream region containing the three motifs had been excised, one in which all three motifs had been mutated, and a further three in which only one of the three motifs had been mutated in turn (Figure 7). We injected the constructs into the gonads of young adult worms and observed GFP expression in the F1 progeny. The construct containing the complete upstream region of *rpl-2* displayed strong GFP expression across most tissues in *C. elegans*, which is consistent with the role of this gene as an essential housekeeping gene. We quantified the level of GFP signal intensity of four to seven different adult *C. elegans* carrying each construct and plotted them relative to the GFP signal intensity of the construct containing the intact upstream region. The constructs in which all three motifs had been excised, all three motifs had been mutated, or only motif 12–5 was mutated all displayed severely reduced GFP expression. The constructs in which only 12–11 or 12–0 were mutated displayed moderately reduced GFP expression.

### Impact of the expression of ***pop-1*** on *rpl-2*

**Expression of the transcription factor *pop-1 *was necessary for the full expression of *rpl-2.*** Given that the absence of motif 12–5 had the greatest impact on the expression of *rpl-2*, and that motif 12–5 is similar to the binding site of *Drosophila* Pan, which is an orthologue of *C. elegans* POP-1, we asked whether the absence of POP-1 might directly impact the expression of *rpl-2*. In order to investigate this question, we used RNAi to knockdown the expression of *pop-1* and then measured the expression level of *rpl-2* by RT-PCR. We found that RNAi knockdown of *pop-1* reduced the expression of *rpl-2* by more than 50% (Additional file 5).

Three binding sites of POP-1 have been described, one in the upstream region of *end-1*, and two in the upstream region of *ceh-22*[38,39]. Although all three sites are within 700 bp of the ATG of a transcript of each gene, none of the sites were identified by the ModuleMaster scan as being similar to motif 12–5. This is because motif 12–5 is based on a CCTTTRA consensus sequence, and while all three sites contain the sequence CTTT, none of the three POP-1 binding sites contain the longer consensus sequence (Figure 1).

## Discussion

We found nine distinct over-represented sequence motifs in *C. elegans* cytoplasmic RPG upstream regions. Of the 84 upstream regions, 80 had instances of at least two of the nine motifs. The existence of three of the motifs (A-rich, AT-rich, and Trans-splice) can be explained by phenomena other than TF binding. Motifs A-rich and AT-rich matched the pattern of 10 bp hyper-periodicity of AA/TT dinucleotides that has been shown to be associated with germline expression in *C. elegans*[40]. These two motifs were only slightly over-represented in RPG upstream regions. Instances of A-rich were far more common than instances of AT-rich, most likely because the A-rich motif was made from a combination of four overlapping DME results while the AT-rich motif was compiled from only two DME results, and consequently the definition of the A-rich motif was much more flexible (Table 1; Figure 1).

The relationship between motifs A-rich and AT-rich and nucleosome coverage was very weak. AA/TT dinucleotides with 10-bp periodicity are associated with nucleosome enrichment, while A-blocks and T-blocks (regions with 3 or more sequential A or T) are associated with nucleosome exclusion [41,42]. Given that motifs A-rich and AT-rich can potentially match both of these patterns, it makes sense that the motifs themselves are not predictive of nucleosome enrichment or exclusion.

About half of the instances of motif Trans-splice overlapped annotated trans-splice acceptor sites in RPG upstream regions. The other instances of this motif possessed some degree of sequence similarity with trans-splice acceptor sites and thus were included by the motif discovery program. RPG upstream regions have a higher concentration of trans-splice acceptor sites than upstream regions in general, which explains why the motif discovery program identified this set of sequences as significantly over-represented. However, trans-splice acceptor sites cannot be identified purely by sequence similarity, therefore, this motif is otherwise unimportant.

Excluding motifs A-rich, AT-rich, and Trans-splice, 71 RPGs had instances of at least one of the remaining motifs in their upstream regions. Each motif was associated with a different subset of the RPGs, indicating that while RPGs contained numerous elements in common, there was no single code that regulated the expression of all of these genes. Two of the motifs were not similar to known TF binding sites and appeared to be entirely novel, however, their functions remain unknown. Motif TGAATA appeared upstream of 16 RPGs and appeared upstream of only 85 genes in general, making it both the rarest motif and the motif with the greatest fold enrichment among RPG upstream regions (Table 1). Motif TTTAGG was much more common in the total set of upstream regions, probably because it had fewer conserved bases and was richer in AT bases than TGAATA. Neither of these two motifs displayed any location bias; both were uniformly distributed across all areas of the upstream regions in which they occurred. The genes associated with these motifs did not display any associations with GO categories other than RPG-related GO categories, suggesting that they may be RPG-specific motifs (Additional file 2).

The remaining four motifs (12–0, 12–5, 12–11, and 12–18) were similar to motifs previously described by Sleumer et al. and were named for their corresponding motifs from that publication [16]. In total this analysis rediscovered five of the eight RPG-associated motifs described in Sleumer et al. [16], including Trans-splice (named 12–8 in Sleumer et al. [16]) (Figure 1). The overlap between the results of the two analyses was not unexpected: although the input data of the two analyses were completely different, the overwhelming association between the motifs described in Sleumer et al. [16] and RPGs suggested that the same patterns would be found when the issue was approached from the other side by first isolating the RPG upstream regions and then searching for motifs. The similarity in the outcomes showed that the motif signals were robust and could be detected regardless of the input data or the algorithm parameters. However, some of the motifs from the earlier publication were not rediscovered here – we assume that they were associated with too few genes to be detected by the current method.

Interestingly, these same four motifs were also found to be similar to characterized TF binding sites from *C. elegans*, human, *Drosophila*, and yeast (Figure 1). We used three motif-motif comparison methods to find the similarities (STAMP, Matcompare, and TESS), and found that they produced non-overlapping results. This finding indicates that in spite of the multitude of available methods, accurate and meaningful motif-motif similarity assessment is still an unsolved problem, and substantial benefits can be gained by compiling the results from a variety of methods. All four motifs have at least one candidate binding protein in *C. elegans*. Together these findings provide evidence that the four motifs function as TF binding sites.

The four motifs also displayed a strong location bias: 95% of the instances of the motifs occurred in the region 149–521 bp upstream of the ATG. This finding indicates that the regulatory region for RPGs is highly compact, and agrees with the modENCODE project report that most TF ChIP-Seq peaks lay within 500 bp upstream of the estimated TSS [20]. Fifty-six of the RPG upstream regions contained at least one of motifs 12–0, 12–5, 12–11, and 12–18 within this range. However, only 34 of the regions contained two of the four motifs, only 12 had at least three, and only one had all four. The small number of RPG upstream regions containing many different putative TF-binding motifs may suggest that all four sites are not necessary for regulation of the RPGs. Conversely, it may simply indicate that our definition of the motifs is too restricted (in order to minimize false positives) and that many binding site locations remain unidentified in this analysis. It would be interesting to determine whether RPG promoters could be distinguished from other types of promoters based solely on the spatial distribution and frequency of the motifs described here, but such an investigation is beyond the scope of the current work.

In spite of the striking similarity between motif 12–0 and the CEH-14 binding site (as determined by ChIP-Seq followed by motif discovery on the resulting peak sequences), it is not certain that CEH-14 is the primary protein that binds to the 12–0 motif. The CEH-14 peaks and instances of motif 12–0 across all upstream regions had a significant amount of overlap: 141 upstream regions both contained an instance of 12–0 and overlapped with a CEH-14 peak (p < 2.2E-16). The CEH-14 peaks were also strongly associated with RPG upstream regions: of the 84 RPG upstream regions, 57 overlapped with a CEH-14 peak (p < 2.2E-16). However, of these 57 CEH-14 peaks, only 23 overlapped with a 12–0 site, which was not significant. The remaining 34 RPG-associated CEH-14 sites did not overlap 12–0 sites in spite of the fact that the general sequence pattern was the same, which may indicate that the definition of the 12–0 site in this analysis may have been too narrow and that many similar sequence locations were not detected.

A confounding fact is that the expression patterns of CEH-14 and RPGs are not related. RPGs display extensive expression in all tissues during embryonic development and cell growth. Conversely, CEH-14 is a LIM homeodomain TF that is exclusively expressed in head and tail neurons, particularly the AFD thermosensory neurons, and is required only for thermotactic behavior [21]. One explanation is that CEH-14 regulates RPG expression only in the few tissues in which it is expressed. However, it seems unlikely that so many RPGs would possess highly conserved regulatory elements for such a narrow range of regulatory control. Another explanation is that the true binding protein to motif 12–0 is a different TF with a similar DNA binding domain. The *C. elegans* genome contains at least seven LIM homeodomain TFs, most of which have uncharacterized binding sites, so it is possible that several of these proteins bind to the same sequences. However, all seven of these TFs display highly specific expression, primarily in individual sensory neurons, motor neurons, and interneurons, so this possibility still does not provide a satisfactory explanation [43]. The modENCODE authors also noted a disconnect between the specific expression of CEH-14 and its observed binding to regions containing a dense collection of TF binding sites, the “highly-occupied target” regions. The authors suggested that this observation could be explained by the existence of another protein that co-binds with CEH-14 to the highly-occupied target regions rather than CEH-14 binding directly [20]. The solution to this conundrum remains to be found. Similarly, the nature of the relationship between motif 12–5 and the transcription factor POP-1 remains unclear. While the RNAi experiment implies that POP-1 is necessary for *rpl-2* expression, the lack of similarity between the three known POP-1 binding sites and the much more highly conserved 12–5 motif suggests that other cofactors may be involved in the interaction.

**We observed a strong co-occurrence of motifs 12–0 and 12–5 in the upstream regions of 250 genes including 22 RPGs (Figure** 2; **Table** 2**).** The motif pair displayed a bias toward two specific relative orientations, which each had a different spatial distribution pattern (Figure 3; Figure 5). Additionally, about half of the motif pairs in the R12-5 ⇛ 12-0 orientation possessed a TACWGTA sequence immediately 5’ to the R12-5 sequence (Figure 6). Taken together, these findings clearly point to an interdependent relationship between the two motifs. Given the evidence that both 12–0 and 12–5 function as TF binding sites (Figure 1), these findings suggest that the proteins that bind to these sites may also bind to each other, or even prevent each other from binding.

Homeodomain-containing TFs have been shown to bind to DNA as monomers, homodimers and heterodimers in a variety of different relative orientations and spacings [44]. Moreover, these TFs may interact with different binding proteins using different regions of the homeodomain depending on their dimerizing partners, which will then also affect the spacing between the two proteins [45]. For example, yeast homeodomain TF MATALPHA2 binds to DNA as a homodimer in several different relative orientations, including on the same or on different strands [46]. The distance between motifs 12–0 and 12–5 was much larger than the space between pairs of homeodomain dimers, but it is comparable to the distance between pairs of Rap1p, Fhl1p, and Tbf1p sites in the upstream regions of yeast RPGs [9]. Although the *C. elegans* motifs described here are not similar to the binding sites of any of the yeast RPG regulators, it is possible that the overall patterns of protein-protein and protein-DNA interactions are related between the two phyla.

The motifs and their RPG enrichment were conserved in other species of genus *Caenorhabditis*, but not in other nematode species (Figure 4). Even *P. pacificus*, which is also a hermaphroditic soil-dwelling nematode, had only a total of 113 instances of motifs 12–0, 12–5, 12–11, 12–18, TGAATA, and TTTAGG among all examined upstream regions, indicating that it does not use these sequences for gene regulation. This finding suggests that investigations of RPG regulation in non-*Caenorhabditis* species could lead to other novel regulatory elements and mechanisms. Additionally, it is consistent with previous findings showing that RPG regulation is often not conserved across species in the same family even though RPGs themselves are coexpressed in all eukaryotes [11], and that regulatory mechanisms evolve much faster than the genes themselves, and throughout evolution, regulatory mechanisms can change while gene expression levels stay the same [9].

While this work was in its final stages of preparation, a paper was published that described a motif pair in the *C. elegans* genome, which was highly similar to the 12–0 ⇛ 12–5 motif pair described here [47]. Our findings of the motifs’ remarkable qualities in terms of relative orientation, co-distribution, location with respect to the ATG of the nearest gene, specificity to genus *Caenorhabditis*, and essentiality for gene expression are directly supported by their observations. The specific definitions of the motifs differed slightly between the two analyses, with the result that the two sets of instances did not overlap exactly: in this work, we found 119 12–0 ⇛ 12–5 motif pairs less than 43 bp apart, while Linhart et al. found 200 similar pairs. The overlap between the two sets of pairs was 50, implying that in total there are at least 269 genes with such a motif pair in their promoters. If the two motif pair definitions were combined for maximum flexibility, perhaps even more instances could be found. Similarly, the work here describes motifs associated with RPGs, while the Linhart et al. analysis focused on genes expressed in germline cells; when combined it is clear that the target genes of this motif pair are part of a superset containing a wide variety of highly expressed and essential genes involved in reproduction, embryonic development, and cell growth.

Linhart et al. stressed the importance of finding other motif pairs that display similar patterns to 12–0 ⇛ 12–5. In this work, we have shown that the R12-5 ⇛ 12–0 relative orientation also possesses an interesting co-distribution, and additionally that a TACWGTA motif occurs directly 5’ to many of its instances. Furthermore, we have discovered four other novel putative TF binding sites, three of which (12–11, 12–18, and TTTAGG) significantly co-occur with motif 12–0. Similarly, Linhart et al. agrees that further experiments are needed to find the TF binding partners of these motifs, and here we have demonstrated the similarities of 12–0 and 12–5 with the CEH-14 ChIP-Seq motif and the POP-1 binding site, respectively, providing immediate candidates.

The 5’ RACE results indicated that the TSS of *rpl-2* was 322 bp upstream of the ATG and only 40 bp downstream of the nearest motif. This was further from the ATG than expected; Kolasinska-Zweirz et al. estimated an average distance of 250 bp between the ATG and the TSS based on H3K4 trimethylation peak data [48]. The motifs’ location relative to the TSS provides further evidence that they are not transcribed and thus most likely function as TF binding sites.

The upstream region of *rpl-2* contains a very short predicted ncRNA, *B0250.15*, which was originally predicted based on a combination of expression, conservation, and RNA secondary structure evidence [20]. The TSS of *rpl-2*, as measured by 5’ RACE, was only 10 bp downstream of the predicted start site of *B0250.15*, which implied that the two genes are co-transcribed and that the ncRNA is generated from the outron (the transcribed section of the pre-mRNA that is removed during trans-splicing), possibly by a similar mechanism to that of mirtrons and intron-contained small ncRNAs [49-51]. Although our experiments have provided further evidence for the transcription of *B0250.15*, there is currently no proof that this gene functions as an independent ncRNA entity; it may simply be a byproduct of the processing of *rpl-2*. Nonetheless, given that 70% of *C. elegans* protein-coding genes are trans-spliced, this finding presents the possibility of a large source of novel ncRNAs [52]. Moreover, a recent investigation of intermediate-sized ncRNAs in *C. elegans* showed that they are enriched in the immediate upstream regions and 5’ UTR introns of protein-coding genes, providing futher candidates for possible outron-produced ncRNAs [53]. Deeper investigation into the locations of the TSSs of trans-spliced genes that are immediately downstream of predicted ncRNAs, and the processing mechanisms of those ncRNAs, could potentially shed light on novel regulatory interactions between outrons, their contained ncRNAs, and their host protein-coding genes.

The current study agrees with recent studies in the aspect that the absence of the motifs resulted in a reduction of gene expression [16,47]. Mutation of the motifs reduced the expression level of the reporter gene in all tissues simultaneously, while deletion of the three motifs obliterated its expression entirely. Mutation of motif 12–5 had the greatest effect; mutation of only four bp within this motif produced a similar reduction of reporter gene expression as that of the mutation of all three motifs at once (Figure 7). However, the mutation of any of the three motifs produced a significant reduction in overall expression levels, showing that they are all necessary for normal expression of *rpl-2*.

## Conclusions

This work has important implications for several fields of research. We have brought to light the unique regulatory system of *C. elegans* RPGs, which consists of seven putative TF binding sites (12–0, 12–5, 12–11, 12–18, TGAATA, TTTAGG, and the R12-5 ⇛ 12–0 flanking sequence), two DNA structural elements as well as the trans-splice acceptor site. Each RPG is regulated by a subset of these elements, showing that the system is highly flexible and allows for a high level of binding site turnover without affecting overall stability. At least four of the elements were part of a larger essential gene regulatory program, while two were specific to RPGs. The elements were not seen in other species outside genus *Caenorhabditis*, which is consistent with previous findings that RPG regulatory systems vary widely among animals, and implies that yet other RPG regulatory elements could be found for each additional nematode species we examine. The reporter gene expression experiments in conjunction with 5’ RACE that we described here can be used to determine the TSSs of other trans-spliced genes, which will provide insight into the potential source of ncRNAs in their currently un-annotated outrons. The co-distribution pattern of 12–0 and 12–5, with its highly specific relative orientation, inter-motif spacing, and flanking sequence, represents a novel arrangement of regulatory elements. Given that such a regulatory element organization has not been seen before, the impact of these findings on our understanding of gene regulation is potentially very large. Determining which proteins bind to these motifs will shed light on the interactions between TFs, their binding sites, and the genes they regulate; the *pop-1* RNAi experiments have already provided a promising candidate for the binding partner of motif 12–5. These findings in turn will have an impact on the fields of systems biology and synthetic biology: every regulatory mechanism we find can greatly expand our understanding of the system as a whole, and these mechanisms can subsequently be used to build new biological systems that perform entirely different functions.

## Methods

### Motif discovery

For each protein-coding transcript in *C. elegans* (total: 22,428), we extracted the region upstream of the ATG to the nearest protein-coding transcript, up to a maximum length of 700 bp and a minimum length of 100 bp (Genome version WS220). Transcripts with different ATGs from the same gene were processed separately. We observed that trans-splice sites were frequently partially or completely obscured by repeat-masking, therefore, we used non-repeat masked DNA. We obtained 81 *C. elegans* cytoplasmic RPGs from the Ribosomal Protein Gene Database [18]. Five RPGs were in downstream positions of operons, but all of them had upstream intercistronic regions longer than 100 bp and were included in the analysis. Three pairs of RPGs were on bidirectional promoters and within 700 bp of each other; one member of each pair was removed from the set. Additionally, six RPGs had two different ATGs, with the result that a total of 84 RPG upstream regions were examined.

We used DME to search for motifs using the set of 84 RPG upstream regions as the foreground and the set of all 22,428 upstream regions as the background [17]. We used a version of DME that did not preface the word-counting step with a repeat-masking step and did not weight motif information content (IC) by base composition. We ran DME at the three different parameter settings (width = 12 bp, IC = 1.5, r = 0.25, g = 0.5, n = 1; width = 12 bp, IC = 1.6, r = 0.25, g = 0.5, n = 1; and width = 14 bp, IC = 1.5, r = 0.25, g = 1.0, n = 1) in an iterative way: after each motif was found, we masked the two central bases of each instance of the motif with Ns and then re-ran DME using the same parameters, up to a total of 20 iterations per parameter set.

We then extracted and merged all motifs that were found at least twice among the results of two different parameter sets. We required an overlap of at least 68% of the instances in the smaller set with those of the larger set in order to merge the two sets into one. During merging, the DME results were aligned and all instances of all motifs were expanded to the maximum width of the aligned result.

We made two exceptions during the motif merge step: One DME result overlapped with 11 instances of TGAATA, but the overlapping region was only eight bp wide, and when we merged it with the other two results for TGAATA, the IC was very low, so we left it out. Another motif had 27 instances, of which 10 overlapped with TGAATA and the other 17 instances overlapped with 12–18, so we left it out as well.

### Plasmid construction and microinjection

We amplified the region 94–671 bp upstream of *rpl-2* (*B0250.1*) from genomic DNA using restriction site overhang primers PB_F and PB_R (Additional file 6). The PCR product was digested with HindIII and BamHI and then cloned into the promoterless GFP-containing vector pPD95-77 (kindly provided by Andrew Fire; Fire Lab *C. elegans* Vector Kit 1995; Addgene plasmid 1495).

We constructed the motif deletion and mutation plasmids by fusion PCR from the previous PCR product [54]. For the deletion plasmid (PB_del), the region containing the three motifs was removed, and for the mutation plasmids, the three motifs were mutated individually (PB_mut_0, PB_mut_5 and PB_mut_11 mutating motifs 12–0, 12–5, and 12–11 respectively) or all three at once (PB_mut) (Additional file 6). We mixed each plasmid with transformation marker pRF4 [rol-6(su1006), roller phenotype] at 50 ng/ul and injected it into the gonad of young adult worms. Stable transgenic lines were established as previously described [55].

For each plasmid, we used four to seven worms to quantify GFP expression. The images were taken by quantitative microscopy and quantified with ImageJ software. The GFP signal intensity of head and pharynx was measured and then normalized by subtracting the background intensity with ImageJ software.

### 5 RACE and RT-PCR

After isolating total RNA from stable transgenic worms carrying the un-mutated PB plasmid, we digested the RNA with DNase I, dephosphorylated with FastAP™ Thermosensitive Alkaline Phosphatase (Fermentas), decapped with Tobacco Acid Pyrophosphatase (Epicentre), and then ligated to a 5’ RACE adapter. The RNA was reverse transcribed with random hexamer primers and amplified with 5’ RACE primer and pPD95_77_2_R for sequencing (Additional file 6) [56].

We confirmed the result by performing RT-PCR using four different pairs of primers: primers B15_F with pPD95_77_2_R; B15_F with B15_R; 5’ RACE primer with pPD95_77_2_R; and 5’ RACE primer with B15_R. As a negative control, we performed RT-PCR using the same template without adding reverse transcriptase.

### RNAi of ***pop-1***

For *pop-1* RNAi, we used the *pop-1* clone from the whole-genome RNAi feeding library as previously described [57]. The total RNAs were isolated from worms after exposure to RNAi bacteria for at least two days (embryonic lethal phenotype), then subjected to DNase I treatment. The expression levels of *rpl-2* were measured with quantitative RT-PCR based on SYBR Green (TransScript^™^ II Green One-Step qRT-PCR SuperMix; TransGen; Additional file 6). Cycling conditions were 50°C for ten minues (for reverse transcription) and 94°C for four minutes, followed by 40 cycles of 94°C for ten seconds, 60°C for ten seconds, and 72°C for 15 seconds. U6 and *tbg-1* were used to normalize the expression level and the relative expression level was calculated as 2^-∆∆Ct^. At least three biological replicates were performed.

Logos for Figures 3 and 6 were produced by WebLogo [58].

## Competing interests

The authors declare that they have no competing interests.

## Authors' contributions

MCS conceived of the study, performed the bioinformatic analysis, and drafted the manuscript. GW and YW designed the primers and generated the expression constructs. HC performed the microinjections and GW analyzed the GFP expression data. GW and YW performed the 5'RACE experiments. GW performed the RNAi experiments. TX supervised HC. RC supervised GW and YW. MQZ supervised MCS as well as supported and supervised the entire project. All authors read and approved the final manuscript.

## Supplementary Material

Additional file 1 **Motif scan results.** The complete list of all scanned locations of motifs 12–0, 12–5, 12–11, 12–18, TGAATA, and TTTAGG. Click here for file

Additional file 2 **DAVID analysis.** GO term and biochemical pathway analysis of the genes associated with each motif. Click here for file

Additional file 3 **Upstream region of *rpl-2.*** Locations of motifs, primers, and ncRNA gene *B0250.15* in the upstream region of *rpl-2*. Click here for file

Additional file 4 **5’ RACE experiment.** Schematic and result of 5’ RACE experiment that was used to determine the TSS of *rpl-2*. Click here for file

Additional file 5 **RNAi experiment.** Result of RNAi experiment that was used to determine the impact of *pop-1* knockdown on the expression level of *rpl-2*. Click here for file

Additional file 6 Primers. Sequences of all primers used for the GFP expression, 5’ RACE, and RNAi experiments. Click here for file
