# Supplementary material for: Regulatory elements of Caenorhabditis elegans ribosomal protein genes
Source: BMC Genomics. 2012 Aug 28;13:433. doi: 10.1186/1471-2164-13-433 (PMC3575287; doi:10.1186/1471-2164-13-433)
Supplement: Additional file 3 — Upstream region of rpl-2. Locations of motifs, primers, and ncRNA gene B0250.15 in the upstream region of rpl-2. [file 1471-2164-13-433-S3.pdf]

Regulatory Elements of *Caenorhabditis elegans* Ribosomal Protein Genes: Additional File 3

Sequence of the entire 700 bp upstream region of *B0250.1* (*rpl-2*). Locations of primers, motifs, predicted ncRNA *B0250.15*, and other genomic annotations are color-coded as indicated.

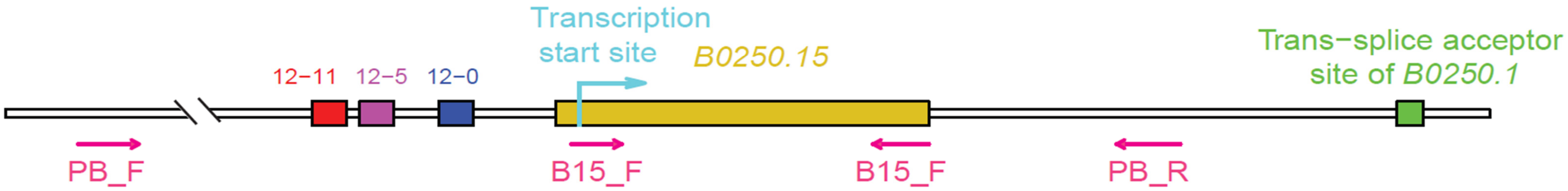

Upstream region of *B0250.1* (*rpl−2*; WBGene00004413)

Upstream region of *B0250.1* (*rpl-2*; WBGene00004413) CHROMOSOME_V:20478964-20479666 (WS220)

TTGCACCCAGATGTTAGTGGTTTAGTGTAGAGAAAGGAGCAGAAGCCGTGAGCCCAGAAGAGACGGCGACAGCTCATCGATTTTTCATTTTCTCTGGCTCTTCTCTCTTTTTCTACGTATTTCTCCG
GTTTTTTAACGATTTTATCAGATTTTCAACCTCAATAATGTTCTGAATAACTTTAAAATCAATTTTTTCTCCAAATTTCAACTTTCTTCCTTCAAAATTCGTAAATTCTTCTCATTTTTCAGATTTAATAT
TAATTTCCCTCTTCTCCTTGTGTCGATTTACGAGAAATGTTAAAGGCGCACGCTATTTATCGGGCTGTCGTGGCGAGACCCACCCTCTCCGCCGCAGCTGCTTGGCTCTCGTTTTCTTCTATTTTTTC
GGCTGGCCTAAACTTTTTTCGCCAATTTTGAAGATTTTCAGTAATTTTTTTTTGTTTTTATGTTCAAATAATTATAGAAATTCAATTTTAATGCTTGTTTTGATCATTCGTCGTCTCTTTCTCTCTCAAAA
TCTCGATTTTTTCGGCTTTTTATCTGAAAAATATTATTTTCCGTCTAAATACGAATTTTTAATTCTCAGTTCCGATAGTTTGTCATCTCTTTCGCTGTTTTACTTTTATTTTCGTCTATTTTTGTAGTGTTT
ACCTCTTTTCCGCAGATTTCAGTCATAATTATTGAATTTTTTCAGGGTAACATG

Motif 12-0
Motif 12-5 (on opposite strand)
Motif 12-11 (on opposite strand)
Sequence of ncRNA *B0250.15*
Trans-splice acceptor site of *B0250.1*
Translation start site of *B0250.1*
Transcription start site as determined by 5’ RACE
PB_F Primer
PB_R Primer (on opposite strand)
