# Supplementary material for: Regulatory elements of Caenorhabditis elegans ribosomal protein genes
Source: BMC Genomics. 2012 Aug 28;13:433. doi: 10.1186/1471-2164-13-433 (PMC3575287; doi:10.1186/1471-2164-13-433)

Regulatory Elements of *Caenorhabditis elegans* Ribosomal Protein Genes: Additional File 4

Top: A schematic of the transcribed product from the plasmid after ligation with the 5’ RACE adapter, and the relative locations of the four primers used in the RT-PCR experiments. Bottom: RT-PCR results. Lane 1 shows the amplification result using primers B15_F and pPD95_77_2_R, which indicates that ncRNA *B0250.15* is co-transcribed with its following sequence (i.e., *B0250.1* in the genome or GFP in the constructed plasmid). Lane 2 shows the amplification result using primers B15_F and B15_R. Lane 3 shows the amplification result using the 5’ RACE primer and pPD95_77_2_R, while lane 4 shows the amplification result using the 5’ RACE primer and B15_R. The distance between the bands in lanes 1 and 2 is similar to the distance between the bands in lanes 3 and 4, which demonstrates the accuracy of the 5’RACE experiment. RT-PCR was also performed using the same template without adding reverse transcriptase as a negative control; no band was produced (data not shown). This result verified that the band was from cDNA amplification and not genomic contamination.

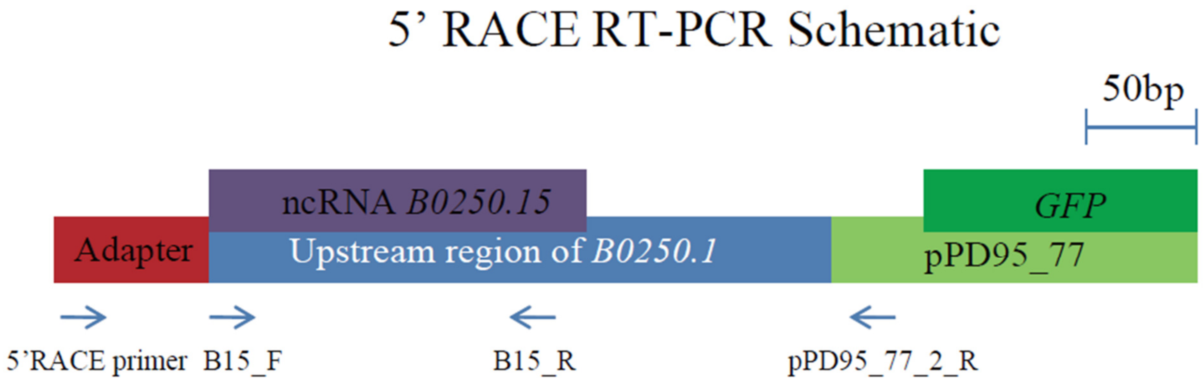

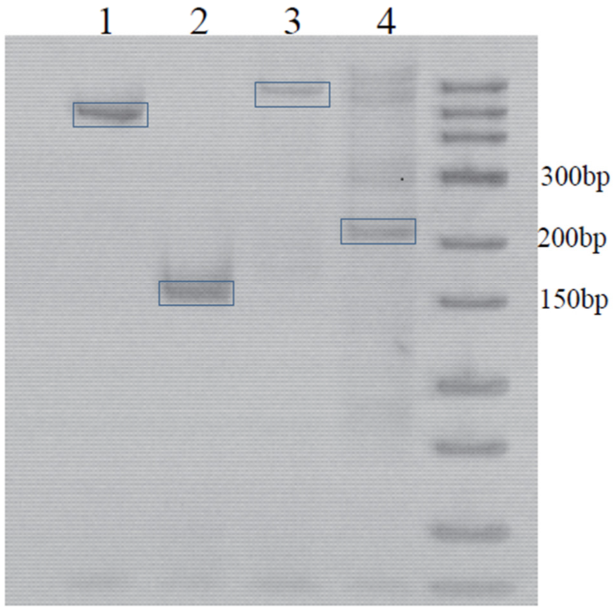

Supplement: Additional file 4 — 5’ RACE experiment. Schematic and result of 5’ RACE experiment that was used to determine the TSS of rpl-2. [file 1471-2164-13-433-S4.pdf]
