# Supplementary material for: Regulatory elements of Caenorhabditis elegans ribosomal protein genes
Source: BMC Genomics. 2012 Aug 28;13:433. doi: 10.1186/1471-2164-13-433 (PMC3575287; doi:10.1186/1471-2164-13-433)

Regulatory Elements of *Caenorhabditis elegans* Ribosomal Protein Genes: Additional File 5

Expression of *rpl-2* following RNAi knockdown of *pop-1*.

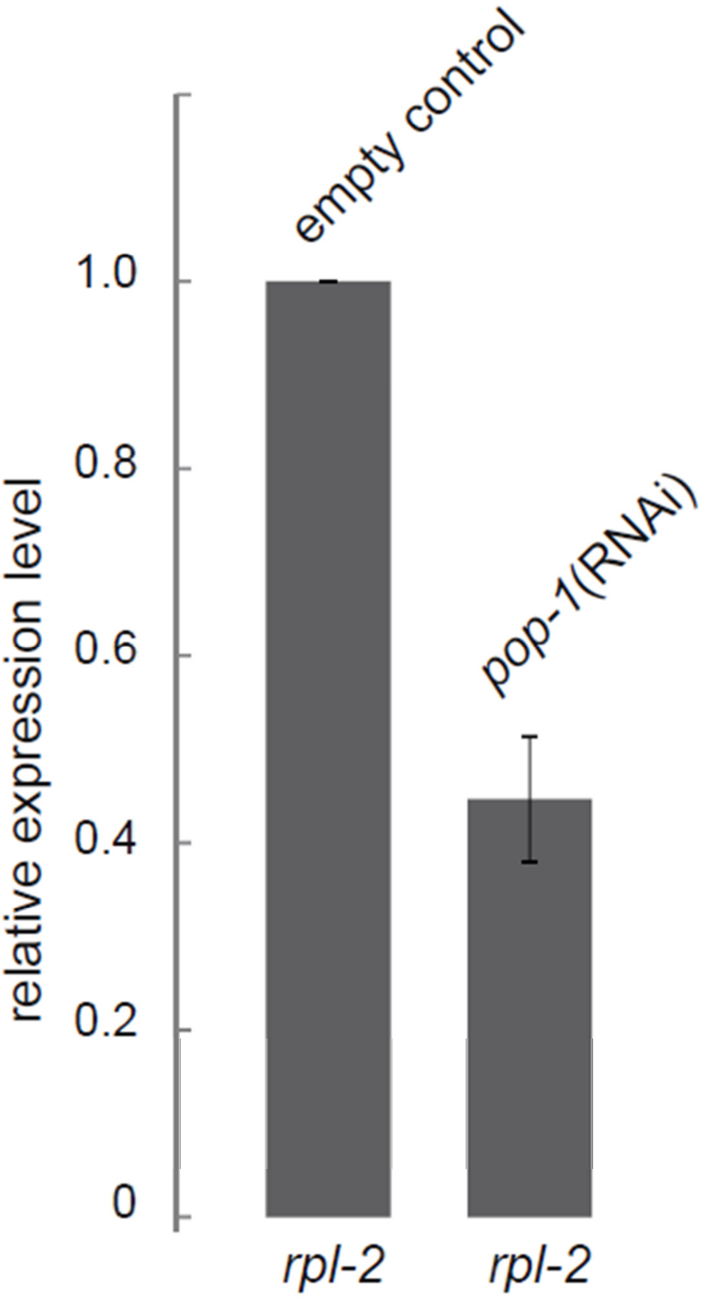

Supplement: Additional file 5 — RNAi experiment. Result of RNAi experiment that was used to determine the impact of pop-1 knockdown on the expression level of rpl-2. [file 1471-2164-13-433-S5.pdf]
