# Supplementary material for: Regulatory elements of Caenorhabditis elegans ribosomal protein genes
Source: BMC Genomics. 2012 Aug 28;13:433. doi: 10.1186/1471-2164-13-433 (PMC3575287; doi:10.1186/1471-2164-13-433)
Supplement: Additional file 6 — Primers. Sequences of all primers used for the GFP expression, 5’ RACE, and RNAi experiments. [file 1471-2164-13-433-S6.pdf]

# Regulatory Elements of *Caenorhabditis elegans* Ribosomal Protein Genes: Additional File 6

The sequences of all primers mentioned in Methods are shown.

| Category | Name | Sequence | Notes |
|---|---|---|---|
| 5’ RACE primers | 5' RACE Adapter | pCATGGCTACATGCTGACAGCCTACTGATGATCAGTCGATGGAAAGGUUUAAUUACCCAAGUUUG | Contains a 5' phosphate. Underlined sequence is RNA. |
| | 5’ RACE Primer | CATGGCTACATGCTGACAGCCTA | |
| | pPD95_77_2_R | GAAAATGTTCTATGTTATGTTAGTATC | |
| | B15_F | TCTATTTTTTCGGCTGGCCT | |
| | B15_R | GAGAGAAAGAGACGACGAATGA | |
| Upstream region of B0250.1 | PB_F | CTGAAGCTTGAGAAAGGAGCAGAAGCCGTGA | Contains HindIII overhang |
| | PB_R | CATGGATCCGAGATGACAAACTATCGGAACTGAG | Contains BamHI overhang |
| PB_del construct primers | PB_del_up_R | AAGGAGAAGAGGGAAATTAATA | All three motifs deleted |
| | PB_del_up_R_down_F | TATTAATTTCCCTCTTCTCCTTCCCTCTCCGCCGCAGC | |
| PB_mut reconstruct primers | PB_mut_F | **GGGT**CGAGAAATGTTA**TTCC**CGCACGCTATTTATCGGGCTGTCGTG**CACT**GACCCA | All three motifs mutated |
| | PB_mut_R | **AGTG**CACGACAGCCCGATAAATAGCGTGCG**GGAA**TAACATTTCTCG**ACCC**TCGACAC | Mutated bases marked with **BOLD** text |
| PB_mut_11 reconstruct primers | PB_mut_11_F | **GGGT**CGAGAAATGTTAAAGGCGCACGCTATTTATCGGGCTGTCGTGGCGAGACCCA | 12-11 mutated |
| | PB_mut_11_R | TCGCCACGACAGCCCGATAAATAGCGTGCGCCTTTAACATTTCTCG**ACCC**TCGACAC | |
| PB_mut_5 reconstruct primers | PB_mut_5_F | TTTACGAGAAATGTTA**TTCC**CGCACGCTATTTATCGGGCTGTCGTGGCGAGACCCA | 12-5 mutated |
| | PB_mut_5_R | TCGCCACGACAGCCCGATAAATAGCGTGCG**GGAA**TAACATTTCTCGTAAATCGACAC | |
| PB_mut_0 reconstruct primers | PB_mut_0_F | TTTACGAGAAATGTTAAAGGCGCACGCTATTTATCGGGCTGTCGTG**CACT**GACCCA | 12-0 mutated |
| | PB_mut_0_R | **AGTG**CACGACAGCCCGATAAATAGCGTGCGCCTTTAACATTTCTCGTAAATCGACAC | |
| qRT-PCR primers | B0250.1.2_F | GTATCCGCATCCAGCGTAAA | *rpl-2* primers |
| | B0250.1.2_R | TTTGCGGTGCTTGTTGTG | |
| | tbg-1_F | CGTCATCAGCCTGGTAGAACA | Control 1 |
| | tbg-1_R | TGATGACTGTCCACGTTGGA | |
| | U6-F | GGAACAATACAGAGAAGATTAGC | Control 2 |
| | U6-R | GGAACGCTTCACGAATTTGC | |
